# Insect–plant relationships predict the speed of insecticide adaptation

**DOI:** 10.1111/eva.13089

**Published:** 2020-08-27

**Authors:** Michael S. Crossley, William E. Snyder, Nate B. Hardy

**Affiliations:** ^1^ Department of Entomology University of Georgia Athens GA USA; ^2^ Department of Entomology and Plant Pathology Auburn University Auburn AL USA

**Keywords:** insecticide resistance, plant–insect interactions, population genetics, pre‐adaptation, survival analysis

## Abstract

Herbivorous insects must circumvent the chemical defenses of their host plants and, in cropping systems, must also circumvent synthetic insecticides. The pre‐adaptation hypothesis posits that when herbivorous insects evolve resistance to insecticides, they co‐opt adaptations against host plant defenses. Despite its intuitive appeal, few predictions of this hypothesis have been tested systematically. Here, with survival analysis of more than 17,000 herbivore–insecticide interactions, we show that resistance evolution tends to be faster when herbivorous insect diets are broad (but not too broad) and when insecticides and plant defensive chemicals are similar (but not too similar). These general relations suggest a complex interplay between macro‐evolutionary contingencies and contemporary population genetic processes, and provide a predictive framework to forecast which pest species are most likely to develop resistance to particular insecticide chemistries.

## 1 INTRODUCTION

For hundreds of millions of years, plants have been evolving chemical defenses against insect herbivores; for just as long, insect herbivores have been evolving to overcome these defenses (Labandeira, 2013). Likewise, for close to a century now in agricultural systems, insect herbivores have been evolving to overcome another set of chemical defenses: synthetic insecticides (Melander, 1914). The pre‐adaptation hypothesis posits that when herbivorous insects evolve resistance to synthetic insecticides, they do so through modification of the same systems used against the chemical defenses of their host plants (Després, David, & Gallet, 2007; Gordon, 1961). Empirical support can be found from instances of cross‐resistance, whereby adaptation of an herbivorous insect population to stout host plant defenses appears to confer increased resistance to insecticides (Dermauw et al., 2013; Zhu, Moural, Nelson, & Palli, 2016). Further support can be found in research linking the breadth of an insect's resistance to insecticides to the breadth of an insect's diet, along with specific diet components (Hardy, Peterson, Ross, & Rosenheim, 2018). Nevertheless, many predictions that could be drawn from the pre‐adaptation hypothesis have yet to be rigorously tested (Hardy et al., 2018). Here, we consider two aspects of herbivore diet history that could influence the rate of resistance evolution: herbivore diet breadth and insecticide‐phytochemical similarity.

The first prediction we derive from the pre‐adaptation hypothesis is that insecticide resistance should evolve faster in insect species with broader diets, that is, diet generalists, since such species will have had evolutionary interactions with more diverse host plant defenses and will have thereby acquired more, or more open‐ended pre‐adaptations for resistance (Ali & Agrawal, 2012; Krieger, Feeny, & Wilkinson, 1971). Note that this prediction runs counter to what one might predict based on the classical population genetics theory, namely that resistance evolution should be faster in diet specialists, as a higher proportion of a specialist population would occur on a crop, and therefore be exposed to selection exerted by insecticides (Georghiou & Taylor, 1986). In fact, the importance of selection frequency is the foundation of refuge‐based approaches for delaying resistance evolution (Carrière et al., 2012; Jin et al., 2015) (although empirical evidence for the efficacy of refuges has come mainly from genetically modified crops). Thus, if we were to observe a positive effect of diet breadth on the rate of resistance evolution, it would suggest that the legacy of diet evolution can override powerful contemporary population genetic forces.

The second prediction we derive from the pre‐adaptation hypothesis is that insecticide resistance should evolve faster when there is a closer similarity between the chemical structure of a pesticide and the phytochemicals that occur in an insect's diet. The premise is that metabolic resistance mechanisms may require only slight modifications to be effective against nonanalog substrates (Berenbaum, Cohen, & Schuler, 1992; Dermauw et al., 2013). That being said, plant secondary metabolites involved in defense often play complex roles in herbivorous insect biology (Scott & Wen, 2001). For example, they can be reused for an herbivore's defense against its own natural enemies (Müller et al., 2001; Smilanich, Fincher, & Dyer, 2016). This complexity raises the possibility of antagonistic pleiotropy for resistance‐conferring alleles: Some evolutionary paths to resistance might come at the expense of disrupting other critical biological functions. Hence, the rate of resistance evolution might increase along with insecticide‐phytochemical similarity only up to a point, beyond which it drops off, as higher chemical similarity begets pleiotropic obstacles to adaptation. If antagonistic pleiotropy is significant, we might expect the highest risk of resistance evolution at some intermediate level of chemical similarity.

To test these predictions, we used mixed‐effect survival model analysis of more than 17,000 specific insect–insecticide interactions.

## 2 MATERIALS AND METHODS

### 2.1 Response variable: Time until resistance evolved

Generic insecticide registration dates for the “all‐in” data set were taken from the Pesticide Properties Database (https://sitem.herts.ac.uk/aeru/ppdb/). For “top‐crops” data, crop‐specific insecticide registration dates (15,183 in all) were obtained from the Agrian label lookup tool (https://home.agrian.com/) and the EPA Pesticide Registration Database (https://iaspub.epa.gov/apex/pesticides/f?p=113:1:::NO:1,5::). In survival analysis, there are two kinds of end points: the occurrence of an event (here, the evolution of insecticide resistance) or the end of the observational period. In the latter case, the observation is right‐censored, that is, set equal to the most recent year of observations, 2020. For the first kind of stop times, we used the earliest reported case of resistance in the Arthropod Pesticide Resistance Database (https://www.pesticideresistance.org/). In 72 cases, the earliest crop‐specific registration date was more recent than the earliest documented case of resistance, usually because resistance preceded the formation of the EPA in 1970. For those cases, we defaulted start times to the generic first‐registration date. For three additional cases, the earliest report of resistance preceded the earliest registration date of any kind. These cases were excluded from the analysis.

### 2.2 Predictor variables

The diet breadth of each herbivorous insect species was characterized as the richness of host plant genera, with data compiled by Hardy et al. (2018). To calculate an index of insecticide‐phytochemical similarity, we began by pulling data on the chemical constituents of plant species from the USDA’s Phytochemical and Ethnobotanical Database (https://phytochem.nal.usda.gov/phytochem/)—in all 30,306 phytochemical‐in‐plant‐species occurrences. The extent of these characterizations varies from one plant species to another, but are especially comprehensive for the top 40 crop species for which we had crop‐specific pesticide registration dates. Next, for each of the 15,215 unique phytochemicals in these characterizations along with the 240 unique insecticides to which at least one species has evolved resistance, we downloaded—if it was available—the canonical SMILES (Simplified Molecular‐Input Line‐Entry System) representation from PubChem (https://pubchem.ncbi.nlm.nih.gov/). These are a simple text‐based specification of chemical structures. They were obtained for 8,416 phytochemicals, a little more than half of the total; excluded were a mix of chemical species such as various alcohols, aldehydes, amino acids, inorganic salts, large proteins, and variants of chemical forms that were successfully characterized. We next calculated all pairwise Tanimoto distances between standard molecular feature fingerprints derived from each SMILES form using the R package RxnSim (Giri, 2017) in R v3.6.2 (R Core Team, 2019). These fingerprints are vectors of hundreds of binary molecular features. For our index of overall similarity between an insecticide and an insect's diet phytochemicals, we used the highest similarity score (which range from zero to one). The all‐in and top‐crops data sets are provided in CSV format as Appendix S1 and S2.

In addition to these two diet‐related predictors (host breadth and phytochemical similarity), we included several covariates that are also likely to affect the rate of resistance evolution. For each species, data on generation time (voltinism) and ploidy (diplodiploidy versus haplodiploidy) were from Rosenheim, Johnson, Mau, Welter, and Tabashnik (1996) and Hardy et al. (2018). In theory, resistance evolution is expected to be faster in haploid species, as heterozygosity cannot shield deleterious alleles from selection (Crowder & Carrière, 2009), and previous empirical evidence indicates that voltinism may have complex effects on rates of adaptation (Rosenheim et al., 1996). As an index of the severity of pest status and documentation bias of each insect species, we used counts of PubMed citations (from now on referred to as “documentation intensity”). We characterized the mode of action of each insecticide according to the classification schema of the Insecticide Resistance Action Committee, filling in missing classifications with data from Pesticide Properties Database.

After removing all incompletely characterized records, the top‐crops data set comprised 332 cases of resistance evolution and 5,305 right‐censored observations (i.e., cases in which resistance has not yet evolved and been documented). The all‐in data set comprised 748 cases of resistance evolution and 16,654 right‐censored observations.

### 2.3 Statistical analysis

We estimated effects of predictor variables on the time until resistance evolved by fitting mixed‐effect proportional hazards models (i.e., Cox models) with the R package coxme (Therneau, 2020b). The main fixed effects were herbivore diet breadth, insecticide‐phytochemical similarity, ploidy, voltinism, and documentation intensity. Diet breadth and documentation intensity were log‐transformed. Diet breadth, documentation intensity, and voltinism were mean‐centered and max–min‐scaled. We did not center and scale insecticide‐phytochemical similarity, as it already ranged from zero to one. In addition to the main fixed effects, we included second‐order polynomial terms for herbivore diet breadth, insecticide‐phytochemical similarity, and voltinism. The model also included as random‐effects insecticide mode of action and a nesting of species identities within taxonomic families. In addition to this proportional hazards model—which was the best fit as measured by AIC—we looked at a variety of alternative parameterizations. Full model specifications are provided as R code in Appendix S3.

In addition to fitting models with the response variable derived from crop‐specific versus generic insecticide registration dates (top‐crops versus all‐in data sets), we took two additional steps to examine the robustness of our estimates. Proportional hazards models assume that fixed effects are proportional, that is, that covariate effects on event hazards do not change over time. Moreover, they assume that hazards are linear combinations of covariate effects. To examine the robustness of our inferences to violations of the assumption of proportionality, we fit additive hazards models (i.e., Aalen's models), which relax the assumption of proportionality, using the R package survival (Therneau, 2020a). Additive hazards models were fit with the same fixed effects as the best‐fitting proportional hazards model, but current implementations are limited to just one random‐effect variable, specified as a so‐called liability term. Thus, we could not incorporate the full random‐effect structure in our main proportional hazards model. Instead, we included as a random‐effect‐only insecticide mode of action, which accounted for the greatest random variation in the proportional hazards model (Table S1). To examine the robustness of our inferences to potential nonlinear interactions between model covariates, we used the R package ranger (Wright & Ziegler, 2017) to conduct a random forests survival analysis. In this approach, complex nonlinear interactions are built‐in, and there is no distinction between fixed and random effects. Because in the ranger implementation there is a limit to the number of states that can be taken by a discrete variable, we could not include a term for herbivore species identity.

## 3 RESULTS

We restricted our analysis to 103 species of North American crop pests for which resistance of any kind has been documented (more than half of which have evolved resistance to multiple insecticides) and for which we could obtain information about several potential risk factors. Our aim was to predict rates of insecticide resistance evolution using survival analysis. At the core of a survival analysis is a hazard function that estimates the risk of an event occurring, in this case, the risk of a particular herbivorous insect species evolving resistance to a particular insecticide. We wanted the outputs of that hazard function to depend on a herbivorous insect's diet breadth, and some measure of the chemical similarity between an insecticide and the phytochemicals in a herbivorous insect's diet, as well as several additional covariates that could affect resistance risk, specifically, insecticide mode of action and a herbivorous insect's generation time, ploidy, severity of pest status, and phylogenetic ancestry.

To characterize how long it took for each case of resistance to evolve, we needed evolutionary start and stop times. For start times, we used pesticide registration dates. In one data set, which we refer to as the “all‐in” data, we used the year at which a pesticide was first registered for use on any crop, and in a second data set, referred to as the “top‐crops” data, and which was restricted to the top 40 crop species (spanning 25 genera) in the United States, we used as evolutionary start times the first year at which an insecticide had been registered for use on a crop genus used by the insect herbivore.

The main features of the fitted hazards models were consistent across top‐crops and all‐in data sets. Here, we share in detail only the results of the crop‐specific (top‐crops) models. We leave a full summary of the all‐in results in Figures S1‐S3 and Tables S1 and S2.

**FIGURE 1:**
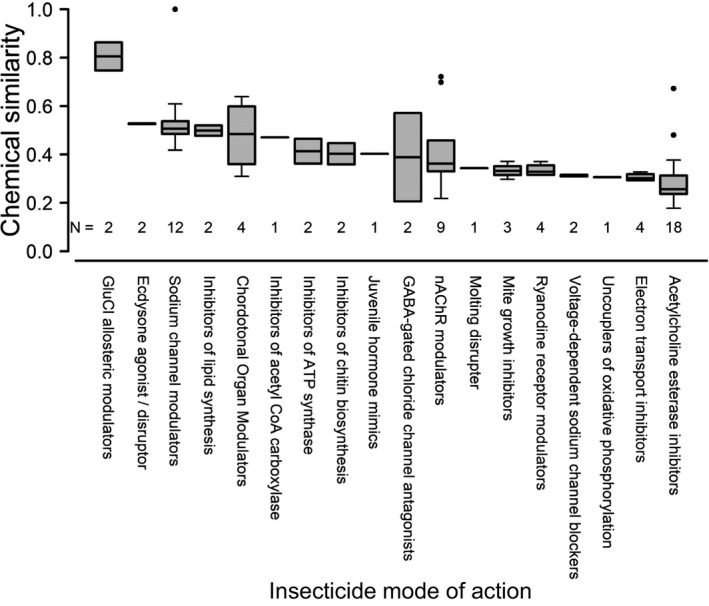
Chemical similarity between insecticides and phytochemicals. Boxplots depict Tanimoto similarity (0 = highly dissimilar; 1 = highly similar) between 72 insecticides and their closest phytochemical analogs, grouped according to insecticide mode of action

Looking at the top‐crops data, each of the 72 examined insecticides was a closest match to one of 55 phytochemicals known to occur in the diets of the insect herbivores, which occur on at least one of our top crops, with maximum insecticide‐phytochemical similarities ranging from 0.18 to 1 (Figure 1). For the all‐in data set, we examined 169 pesticides, which most closely matched one of 1,016 phytochemicals in herbivore diets. Maximum insecticide‐phytochemical similarities ranged from 0.079 to 1. The majority of closest (or identical) insecticide‐phytochemical matches were between synthetic pyrethroids and pyrethrins (sodium channel modulators; Figure 1), well‐known naturally occurring insecticides.

**FIGURE 2:**
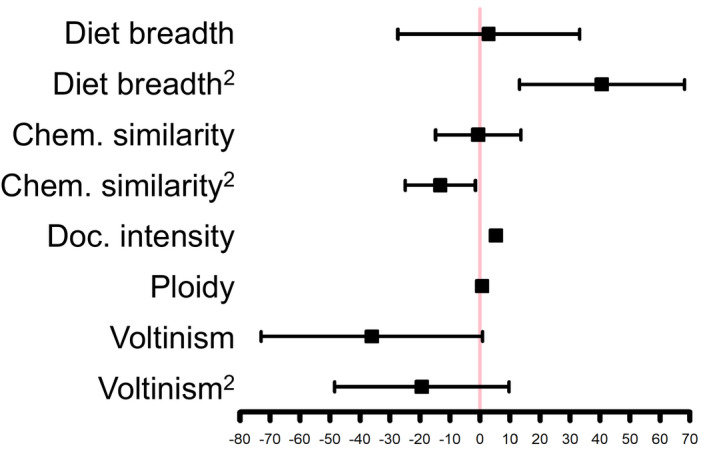
Effects of predictors on probability of insecticide resistance evolution. Forest plots depict top‐crops proportional hazards model risk factors. Boxes denote estimated fixed effects. Whiskers show 95% confidence intervals (+ or − 1.96 * *SE*). The vertical red line denotes an effect of zero

Estimated effects for a proportional hazards model are shown in Figure  2 and summarized in Table S3. Herbivore diet breadth had a U‐shaped effect on the rate of insecticide resistance evolution; the main effect was insignificant, whereas the second‐order effect was significant and positive (coefficient: 40.6; *p*‐value = .004). Thus, the mapping of diet breadths to resistance times was a concave function with no overall increasing or decreasing trend. Insecticide‐phytochemical similarity had a hump‐shaped effect on rates of resistance evolution; the main effect was insignificant, but the second‐order effect was significant and negative (coefficient: −13.2; *p*‐value = .02). Documentation intensity positively covaried with resistance evolution (coefficient: 5.3; *p*‐value < .001). Neither Voltinism nor ploidy had a significant effect.

**FIGURE 3:**
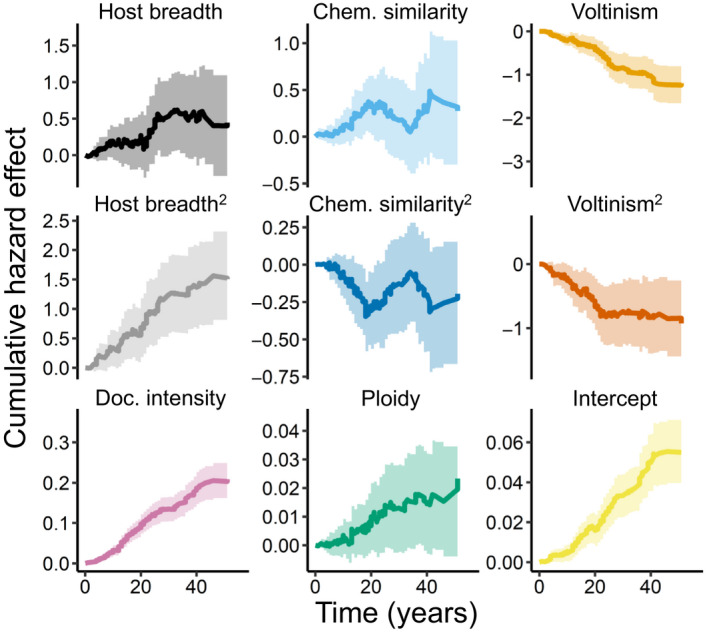
Effects of predictors on probability of insecticide resistance evolution over time. Line plots depict cumulative hazards effects from top‐crops additive hazards model (light shading represents 95% confidence intervals). The intercept plot shows how the baseline risk of insecticide resistance increases over time. The rest of the plots show how each model covariate modify that baseline hazard

Proportional hazards models assume that hazards are constant over time. We also fit an additive hazards model that relaxed this assumption, although at the cost of a less realistic set of random effects. Results of this additive model were largely congruent with those from proportional hazards models (Figure 3, Table S4), but differed in the following ways. In the additive model, the main effect of diet breadth was positive and just short of significant (coefficient: 0.02; *p*‐value = 0.07). Simply put, increasing diet generalism appeared to increasingly elevate the risk of resistance evolution, and there was no realized rate of adaptation advantage for specialists. The first‐ and second‐order effects from voltinism were negative and significant (first‐order coefficient: −0.037; first‐order *p*‐value = .0027; second‐order coefficient: −0.034; second‐order *p*‐value = .0013). Also, in the all‐in model, the main effect of insecticide‐phytochemical similarity was positive and significant (coefficient: 0.027; *p*‐value 0.0035), meaning that there was an overall increase in risk from chemical similarity. To repeat, this additive hazards model relaxes the assumption that effects are constant over time, but has a less complex set of random effects.

**FIGURE 4:**
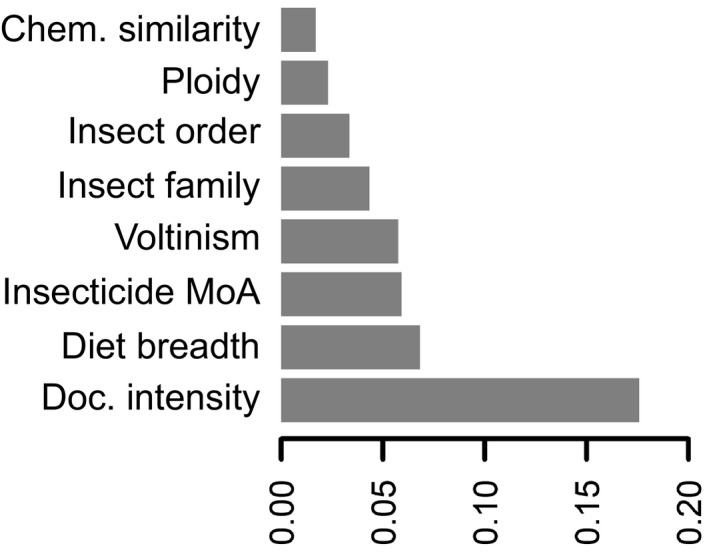
Top‐crops random forest survival model variable importance

The relative importance of predictor variables in a random forests survival analysis is shown in Figure 4. This ranking is consistent across the top‐crops and all‐in data sets (Figure S3). The prediction error (1 – Harrell's concordance index) was ~18%. This error was ~15% for the all‐in data set. In a random forest analysis, one does not estimate variable coefficients per se, but we can get a sense for the overall congruence between the random forest survival analysis and the proportional hazards model analysis by looking at the correlation between predictions of resistance evolution rates, for each herbivore–insecticide combination. Pearson's coefficient for the correlation between predictions was 85% (*p*‐value < 2.2e‐16).

## 4 DISCUSSION

Does an herbivorous insect's evolutionary diet history predict the rate of insecticide adaptation? It seems so, although in complex ways. We tested two main predictions. The first was that the risk of resistance evolution would depend on an herbivore's diet breadth. Following the pre‐adaptation hypothesis, we expected faster evolution of resistance in generalists, as generalists should have more pre‐adaptive capacity (Dermauw et al., 2013; Krieger et al., 1971). On the other hand, following classical population genetics theory, we expected faster evolution of resistance in specialists, as specialists would be more often exposed to selection from insecticide applications on specific crops (Georghiou & Taylor, 1986). What we found was evidence in support of both predictions. Resistance evolution was faster in the most specialized species, and in the most generalist. Thus, we have indirect evidence for the importance of population genetic features affecting the efficiency of selection, as well as the potential for macro‐evolutionary history to override those population genetic forces by shaping the pre‐adaptive capacity of populations.

The second main idea we sought to test was that the rate of resistance evolution would depend on the chemical similarity between a particular insecticide and the phytochemicals that occur in an herbivore's diet. Following the pre‐adaptation hypothesis, we predicted faster resistance evolution when insecticides and phytochemicals were more similar in chemical structure. If herbivorous insects overcome insecticides with adjustments to systems to overcome plant defenses (Després et al., 2007; Gordon, 1961), a close similarity between plant defenses and insecticides could mean that relatively small adjustments would suffice. On the other hand, since the secondary metabolites that are used in plant defense often play multifarious roles in herbivorous insect biology (Raubenheimer & Simpson, 2009; Scott & Wen, 2001), we expected that high insecticide‐phytochemical similarity could impede resistance adaptation via antagonistic pleiotropy. Here again, we found evidence supporting both predictions. The risk of resistance evolution increased with increasing insecticide‐phytochemical similarity up to a point and then dropped off toward the highest similarities. As for diet breadth, we found that the risk of resistance evolution is determined by both the legacy of diet history and contemporary genetic constraints on adaptation.

We have stressed the results of the mixed‐effect proportional hazards model (versus the additive hazards model), as it is the model that most fully accounts for the potentially confounding factors of phylogenetic coancestry and variation in insecticide modes of action. The influential predictors were largely the same across models, but the shape of some relationships differed. Specifically, in the additive hazards models, increases across the full range of diet breadths were linked to increases in resistance evolution risk (Figure 3 and Figure S2). Thus, if we relax the assumption that risks have been constant over time, and use a simpler (and less realistic) random variable structure, pre‐adaptive capacity (greater diet generalism) would seem to be even more important than contemporary population genetic forces (acting disproportionately on diet specialists) in governing the risk of resistance evolution. Another difference was that in the additive hazards model of the all‐in data, the risk of resistance evolution increased monotonically with increasing insecticide‐phytochemical similarity. As a reminder, in the proportional hazards model, the effect was hump‐shaped. In this case, the differences in fixed effects could be due to changes in the random‐effects structure; if we drop the taxonomic random effects from the proportional hazards model (leaving only random effects from insecticide mode of action), we also find a positive monotonic relationship. The additive hazards model also found a significant convex effect of voltinism on the risk of resistance evolution. This is consistent with results from previous work (Hardy et al., 2018; Rosenheim et al., 1996), but so far has eluded explanation. Here, we treat voltinism as a nuisance parameter and refrain from interpretation. The same goes for ploidy. In the additive hazards models, haplodiploidy caused a small but significant increase in the risk of resistance evolution. This is consistent with theory: In haploids, selection against insecticide‐susceptible alleles is stronger, as they cannot be masked by heterozygosity (Crowder & Carrière, 2009).

To justify our predictions of how host‐use history might affect rates of resistance evolution, we focused on metabolic resistance. Another common mode of resistance is target site insensitivity, where a mutation causes a structural change in the vicinity of an insecticide's binding site—for example, on a sodium‐ion channel protein or a neural modulator receptor (Sparks & Nauen, 2015)—that reduces binding efficiency. Target site insensitivity is also a means by which herbivorous insects evolve to resist host plant defenses (e.g., Zhen, Aardema, Medina, Schumer, & Andolfatto, 2012). Thus, insofar as insecticides and plant defensive chemicals share binding sites, the pre‐adaptation hypothesis could also be used to predict insecticide resistance via target site insensitivity. But the specific predictions may differ from those we made for metabolic resistance; for example, target site insensitivity to plant defensive chemicals appears to be more common in herbivores with more specialized diets (Zhen et al., 2012).

Though our study presents a novel integration of diverse data to address a fundamental question in evolutionary biology, we acknowledge several shortcomings. First, our estimates of insecticide‐phytochemical similarity were challenged by the difficulty of modeling the structure of many of the phytochemicals and determining biologically relevant shared features (Maggiora, Vogt, Stumpfe, & Bajorath, 2014), as well as the uneven intensity at which host plant secondary chemistries have been characterized. This may have contributed to the relatively low importance of phytochemical similarity in the random forests survival analysis (Figure 4). Second, survival models are seldom used in evolutionary ecology research, and current implementations offer limited means of incorporating phylogenetic coancestries as random effects. Lastly, as in almost any ecological study, we have explicitly incorporated only few of the potential variables that could in some way affect resistance evolution. Our model construction was guided mainly by expediency. Thus, the significant effects that we identified might not be directly causal. In fact, they are almost definitely not. For example, when we predict that a broad diet will increase the risk of resistance evolution, diet breadth itself encompasses several latent genetic, physiological, and population genetic variables that are more directly related to the evolution of insecticide resistance. Examples of such latent variables are the diversity and substrate specificity of detoxification enzymes, the modularity of gene expression networks, and the standing genetic diversity in populations (Hardy, Kaczvinsky, Bird, & Normark, 2020). Hence, the high rates of resistance evolution among diet generalists should be interpreted as denoting the ultimate importance of yet‐to‐be characterized differences in the biology between generalists and specialists.

In this study, we used survival models primarily to explain variation in the time it took for herbivorous insect populations to evolve resistance to insecticides. The same models can also be used to predict future cases of resistance. For example, according to our models, the highest risks are for a few major pests (*Heliothis virescens*, *Leptinotarsa decemlineata*, *Myzus persicae*, *Plutella xylostella*) evolving resistance to mostly pyrethroid insecticides (Tables S5 and S6). Perhaps it comes as no surprise that the highest risks are for some of the most notorious pest species, and to one of the most often‐resisted insecticide classes. But it is our hope that these predictions will help direct management strategies to forestall resistance evolution. Indeed, our predictions cover likely cases of future resistance for each of the 103 pest species considered. Thus, the potential for shaping resistance management strategies is broad.

## 5 CODE AVAILABILITY STATEMENT

R code used to curate and analyze data is available in Appendix S3.

## CONFLICT OF INTERESTS

The authors declare no competing interests.

## AUTHOR CONTRIBUTIONS

N.B.H., M.S.C., and W.E.S. conceived of the idea for the paper and contributed to writing the manuscript. N.B.H conducted analyses, and M.S.C. assisted with data collection and visualization.

## Supporting information

Supplementary Material

## Data Availability

Data supporting the findings of this study (the “all‐in” and “top‐crops” data sets) are available and are provided in CSV format in Appendix S1 and S2.

## References

[eva13089-bib-0001] Ali, J. G. , & Agrawal, A. A. (2012). Specialist versus generalist insect herbivores and plant defense. Trends in Plant Science, 17(5), 293–302. 10.1016/j.tplants.2012.02.006 22425020

[eva13089-bib-0002] Berenbaum, M. R. , Cohen, M. B. , & Schuler, M. A. (1992). Cytochrome P450 monooxygenase genes in oligophagous Lepidoptera In: C. A. Mullin , & J. G. Scott (Eds.), ACS Symposium Series 505 (pp 114–124). Washington, DC: American Chemical Society 10.1021/bk-1992-0505.ch009

[eva13089-bib-0003] Carriere, Y. , Ellers‐Kirk, C. , Hartfield, K. , Larocque, G. , Degain, B. , Dutilleul, P. , … Tabashnik, B. E. (2012). Large‐scale, spatially‐explicit test of the refuge strategy for delaying insecticide resistance. Proceedings of the National Academy of Sciences of the United States of America, 109(3), 775–780. 10.1073/pnas.1117851109 22215605 PMC3271916

[eva13089-bib-0004] Crowder, D. W. , & Carrière, Y. (2009). Comparing the refuge strategy for managing the evolution of insect resistance under different reproductive strategies. Journal of Theoretical Biology, 261(3), 423–430. 10.1016/j.jtbi.2009.08.017 19703471

[eva13089-bib-0005] Dermauw, W. , Wybouw, N. , Rombauts, S. , Menten, B. , Vontas, J. , Grbic, M. , … Van Leeuwen, T. (2013). A link between host plant adaptation and pesticide resistance in the polyphagous spider mite Tetranychus urticae. Proceedings of the National Academy of Sciences, 110(2), E113–E122. 10.1073/pnas.1213214110 PMC3545796 23248300

[eva13089-bib-0006] Després, L. , David, J. P. , & Gallet, C. (2007). The evolutionary ecology of insect resistance to plant chemicals. Trends in Ecology & Evolution, 22(6), 298–307. 10.1016/j.tree.2007.02.010 17324485

[eva13089-bib-0007] Georghiou, G. , & Taylor, C. (1986). Factors influencing the evolution of resistance In N. R. Council (Ed.), Pesticide Resistance: Strategies and Tactics for Management (pp. 157–169). Washington, D.C.: National Academy Press.

[eva13089-bib-0008] Giri, V. (2017). RxnSim: Functions to Compute Chemical Reaction Similarity. Retrieved from https://cran.r‐project.org/package=RxnSim

[eva13089-bib-0009] Gordon, H. T. (1961). Nutritional factors in insect resistance to chemicals. Annual Review of Entomology, 6(1), 27–54. 10.1146/annurev.en.06.010161.000331

[eva13089-bib-0010] Hardy, N. B. , Kaczvinsky, C. , Bird, G. , & Normark, B. B. (2020). What we don’t know about the evolution of diet breadth in herbivorous insects. Annual Reviews in Ecology Evolution and Systematics, 51, 103‐122. 10.1146/annurev-ecolsys-011720-023322

[eva13089-bib-0011] Hardy, N. B. , Peterson, D. A. , Ross, L. , & Rosenheim, J. A. (2018). Does a plant‐eating insect’s diet govern the evolution of insecticide resistance? Comparative tests of the pre‐adaptation hypothesis. Evolutionary Applications, 11, 739–747. 10.1111/eva.12579 29875815 PMC5979754

[eva13089-bib-0012] Jin, L. , Zhang, H. , Lu, Y. , Yang, Y. , Wu, K. , Tabashnik, B. E. , & Wu, Y. (2015). Large‐scale test of the natural refuge strategy for delaying insect resistance to transgenic Bt crops. Nature Biotechnology, 33(2), 169–174. 10.1038/nbt.3100 25503384

[eva13089-bib-0013] Krieger, R. I. , Feeny, P. P. , & Wilkinson, C. F. (1971). Detoxication enzymes in the guts of caterpillars: An evolutionary answer to plant defenses? Science, 172(3983), 579–581. 10.1126/science.172.3983.579 5555079

[eva13089-bib-0014] Labandeira, C. C. (2013). A paleobiologic perspective on plant–insect interactions. Current Opinion in Plant Biology, 16(4), 414–421. 10.1016/j.pbi.2013.06.003 23829938

[eva13089-bib-0015] Maggiora, G. , Vogt, M. , Stumpfe, D. , & Bajorath, J. (2014).Molecular similarity in medicinal chemistry. Journal of Medicinal Chemistry, 57(8), 3186–3204. 10.1021/jm401411z 24151987

[eva13089-bib-0016] Melander, A. L. (1914). Can insects become resistant to sprays? Journal of Economic Entomology, 7(2), 167–173. 10.1093/jee/7.2.167

[eva13089-bib-0017] Müller, C. , Agerbirk, N. , Olsen, C. E. , Boevé, J. L. , Schaffner, U. , & Brakefield, P. M. (2001). Sequestration of host plant glucosinolates in the defensive hemolymph of the sawfly *Athalia rosae* . Journal of Chemical Ecology, 27(12), 2505–2516. 10.1023/A:1013631616141 11789955

[eva13089-bib-0018] R Core Team (2019). R: A Language and Environment for Statistical Computing. Vienna, Austria: R Core Team Retrieved from https://www.r‐project.org/

[eva13089-bib-0019] Raubenheimer, D. , & Simpson, S. J. (2009). Nutritional PharmEcology: Doses, nutrients, toxins, and medicines. Integrative and Comparative Biology, 49(3), 329–337. 10.1093/icb/icp050 21665823

[eva13089-bib-0020] Rosenheim, J. A. , Johnson, M. W. , Mau, R. F. L. , Welter, S. C. , & Tabashnik, B. E. (1996). Biochemical preadaptations, founder events, and the evolution of resistance in arthropods. Journal of Economic Entomology, 89, 263–273.

[eva13089-bib-0021] Scott, J. G. , & Wen, Z. (2001). Cytochromes P450 of insects: The tip of the iceberg. Pest Management Science, 57, 958–967. 10.1002/ps.354 11695190

[eva13089-bib-0022] Smilanich, A. M. , Fincher, R. M. , & Dyer, L. A. (2016). Does plant apparency matter? Thirty years of data provide limited support but reveal clear patterns of the effects of plant chemistry on herbivores. New Phytologist, 210(3), 1044–1057. 10.1111/nph.13875 26889654

[eva13089-bib-0023] Sparks, T. C. , & Nauen, R. (2015). IRAC: Mode of action classification and insecticide resistance management. Pesticide Biochemistry and Physiology, 121, 122–128. 10.1016/j.pestbp.2014.11.014 26047120

[eva13089-bib-0024] Therneau, T. M. (2020a). A Package for Survival Analysis in R. Retrieved from https://cran.r‐project.org/package=survival

[eva13089-bib-0025] Therneau, T. M. (2020b). coxme: Mixed Effects Cox Models. Retrieved from https://cran.r‐project.org/package=coxme

[eva13089-bib-0026] Wright, M. N. , & Ziegler, A. (2017). Ranger: a fast implementation of random forests for high dimensional data in C++ and R. Journal of Statistical Software, 77(1), 1–17. 10.18637/jss.v077.i01

[eva13089-bib-0027] Zhen, Y. , Aardema, M. L. , Medina, E. M. , Schumer, M. , & Andolfatto, P. (2012). Parallel molecular evolution in an herbivore community. Science, 337(6102), 1634–1637. 10.1126/science.1226630 23019645 PMC3770729

[eva13089-bib-0028] Zhu, F. , Moural, T. W. , Nelson, D. R. , & Palli, S. R. (2016). A specialist herbivore pest adaptation to xenobiotics through up‐regulation of multiple cytochrome P450s. Scientific Reports, 6(1), 20421. doi: 10.1038/srep20421 26861263 PMC4748221

