# Supplementary material for: Insect–plant relationships predict the speed of insecticide adaptation
Source: Evol Appl. 2020 Aug 27;14(2):290–6. doi: 10.1111/eva.13089 (PMC7896708; doi:10.1111/eva.13089)
Supplement: Supplementary file 1 — Supplementary Material [file EVA-14-290-s001.zip › eva13089-sup-0005-Supinfo.docx]

Supplementary Information

**Supplementary Table 1.** All-in proportional hazard model effects. Cox mixed-effects model: Surv(delta, status) ~ poly(host_count, 2) + poly(chemdist, 2) + docs + ploidy + poly(gpy, 2) + (1 | Family/Species) + (1 | MoA).

| *Fixed effects* | **Group** | **coefficient** | **exp(coef.)** | **se(coef.)** | **z** | **p** |
| --- | --- | --- | --- | --- | --- | --- |
|  | Diet breadth | 11.4 | 9.2E-04 | 20.6 | 0.56 | 0.58 |
|  | Diet breadth^2^ | 47.5 | 4.1E+20 | 23.4 | 2.03 | **0.04** |
|  | Chemical similarity | -5.9 | 2.7E-03 | 6.8 | -0.87 | 0.38 |
|  | Chemical similarity^2^ | -21.1 | 6.7 E-10 | 5.6 | -3.78 | **1.6E-04** |
|  | Documentation intensity | 3.3 | 27.3 | 0.5 | 6.97 | **3.1E-12** |
|  | Ploidy | 0.3 | 1.4 | 0.3 | 1.05 | 0.29 |
|  | Voltinism | -22.1 | 2.5E-10 | 17.6 | -1.26 | 0.21 |
|  | Voltinism^2^ | -22.7 | 1.3E-10 | 19.9 | -1.15 | 0.25 |
|  |  |  |  |  |  |  |
| *Random effects* | **Group** | **Variable** | **Std. dev.** | **Variance** |  |  |
|  | Family/Species | Intercept | 0.76 | 0.58 |  |  |
|  | Family | Intercept | 0.23 | 0.05 |  |  |
|  | Insecticide mode of action | Intercept | 0.83 | 0.68 |  |  |

**Supplementary Table 2.** All-in additive hazard model effects. Aalen’s model fit: Surv(delta, status) ~ docs + poly(chemdist, 2)+ poly(host_count, 2)+ ploidy + poly(gpy, 2)+ frailty(MoA).

| **Group** | **slope** | **coefficient** | **se(coef.)** | **z** | **p** |
| --- | --- | --- | --- | --- | --- |
| Intercept | 1.1E-03 | 4.9E-05 | 3.5E-06 | 14.00 | **1.2E-44** |
| Documentation intensity | 5.3E-03 | 2.2E-04 | 1.2E-05 | 18.90 | **1.8E-79** |
| Chemical similarity | 2.7E-02 | 1.0E-03 | 3.5E-04 | 2.92 | **3.5E-03** |
| Chemical similarity^2^ | -3.4E-02 | -1.2E-03 | 2.5E-04 | -4.95 | **7.3E-07** |
| Diet breadth | 5.3E-02 | 1.9E-03 | 4.0E-04 | 4.70 | **2.6E-06** |
| Diet breadth^2^ | 1.1E-01 | 4.2E-03 | 5.1E-04 | 8.26 | **1.4E-16** |
| Ploidy | 9.3E-04 | 4.0E-05 | 6.4E-06 | 6.25 | **4.1E-10** |
| Voltinism | -3.3E-02 | -1.1E-03 | 2.8E-04 | -4.12 | **3.8E-05** |
| Voltinism^2^ | -6.9E-02 | -2.4E-03 | 3.9E-04 | -6.17 | **6.7E-10** |
| frailty(Insecticide Mode of Action) | 2.9E-05 | 9.8E-07 | 4.3E-07 | 2.26 | **2.4E-02** |

**Supplementary Table 3.** Top-crops proportional hazard model effects. Cox mixed-effects model: Surv(delta, status) ~ poly(host_count, 2) + poly(chemdist, 2) + docs + ploidy + poly(gpy, 2) + (1 | FAMILY/sp) + (1 | MoAl2).

| *Fixed effects* | **Group** | **coefficient** | **exp(coef.)** | **se(coef.)** | **z** | **p** |
| --- | --- | --- | --- | --- | --- | --- |
|  | Diet breadth | 2.9 | 1.8E+01 | 15.5 | 0.19 | 0.85 |
|  | Diet breadth^2^ | 40.6 | 4.5E+17 | 14.1 | 2.89 | **0.004** |
|  | Chemical similarity | -0.6 | 0.6 | 7.3 | -0.08 | 0.93 |
|  | Chemical similarity^2^ | -13.2 | 1.8E-06 | 5.9 | -2.22 | **0.02** |
|  | Documentation intensity | 5.3 | 205.6 | 0.8 | 6.66 | **2.7E-11** |
|  | Ploidy | 0.7 | 2.1 | 0.5 | 1.51 | 0.13 |
|  | Voltinism | -36.1 | 2.1E-16 | 18.8 | -1.92 | 0.06 |
|  | Voltinism^2^ | -19.4 | 3.9E-09 | 14.8 | -1.31 | 0.19 |
|  |  |  |  |  |  |  |
| *Random effects* | **Group** | **Variable** | **Std. dev.** | **Variance** |  |  |
|  | Family/Species | Intercept | 0.98 | 0.96 |  |  |
|  | Family | Intercept | 0.36 | 0.13 |  |  |
|  | Insecticide Mode of Action | Intercept | 0.95 | 0.91 |  |  |

**Supplementary Table 4.** Top-crops additive hazard model effects. Aalen’s model fit: Surv(delta, status) ~ docs + poly(chemdist, 2)+ poly(host_count, 2)+ ploidy + poly(gpy, 2)+ frailty(MoAl2).

| **Group** | **slope** | **coefficient** | **se(coef.)** | **z** | **p** |
| --- | --- | --- | --- | --- | --- |
| Intercept | 1.5E-03 | 2.7E-04 | 3.8E-05 | 7.08 | **1.5E-12** |
| Documentation intensity | 6.9E-03 | 1.1E-03 | 1.1E-04 | 10.10 | **7.8E-24** |
| Chemical similarity | 1.9E-02 | 1.9E-03 | 1.5E-03 | 1.30 | 0.19 |
| Chemical similarity^2^ | -1.1E-02 | -1.5E-03 | 1.1E-03 | -1.29 | 0.19 |
| Diet breadth | 2.1E-02 | 3.2E-03 | 1.8E-03 | 1.77 | 0.07 |
| Diet breadth^2^ | 5.7E-02 | 8.4E-03 | 2.1E-03 | 3.96 | **7.4E-06** |
| Ploidy | 6.3E-04 | 1.0E-04 | 5.2E-05 | 1.97 | **0.049** |
| Voltinism | -3.7E-02 | -6.4E-03 | 1.2E-03 | -5.58 | **2.4E-08** |
| Voltinism^2^ | -4.2E-02 | -5.5E-03 | 1.7E-03 | -3.26 | **1.1E-03** |
| frailty(Insecticide Mode of Action) | -2.8E-06 | -3.3E-06 | 3.4E-06 | -0.96 | 0.34 |

**Supplementary Table 5.** Predicted proportional hazards of arthropods and insecticides according to the “top-crops” model, expressed in terms of expected number of cases of resistance per year.

**Supplementary Table 6.** Predicted proportional hazards of arthropods and insecticides according to the “all-in” model, expressed in terms of expected number of cases of resistance per year.

**
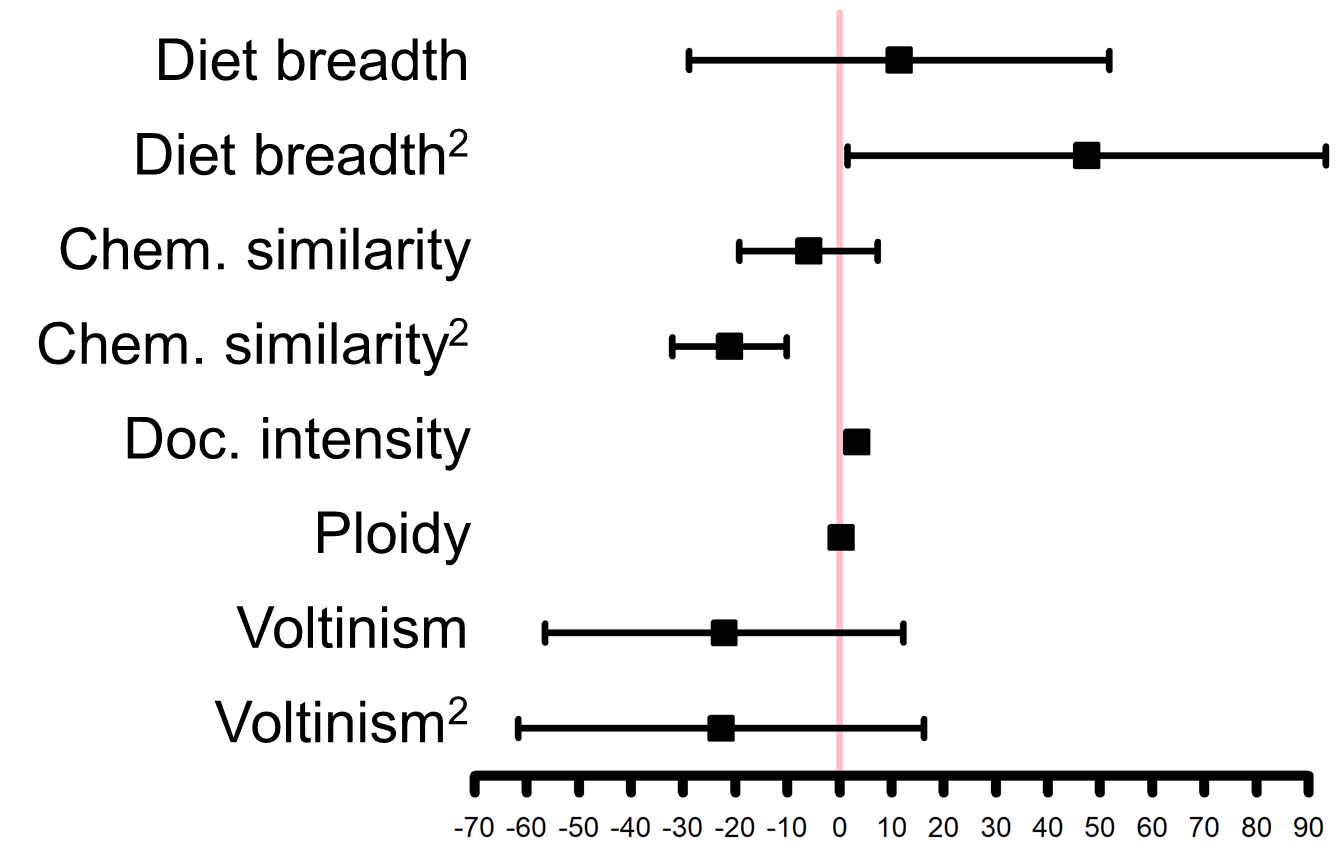
**

**Supplementary Figure 1.** **Effects of predictors on probability of insecticide resistance evolution.** Forest plots depict all-in proportional hazard model risk factors. Boxes denote estimated fixed effects. Whiskers show 95% confidence intervals (+ or – 1.96 * standard error). The vertical red line denotes an effect of zero.

**
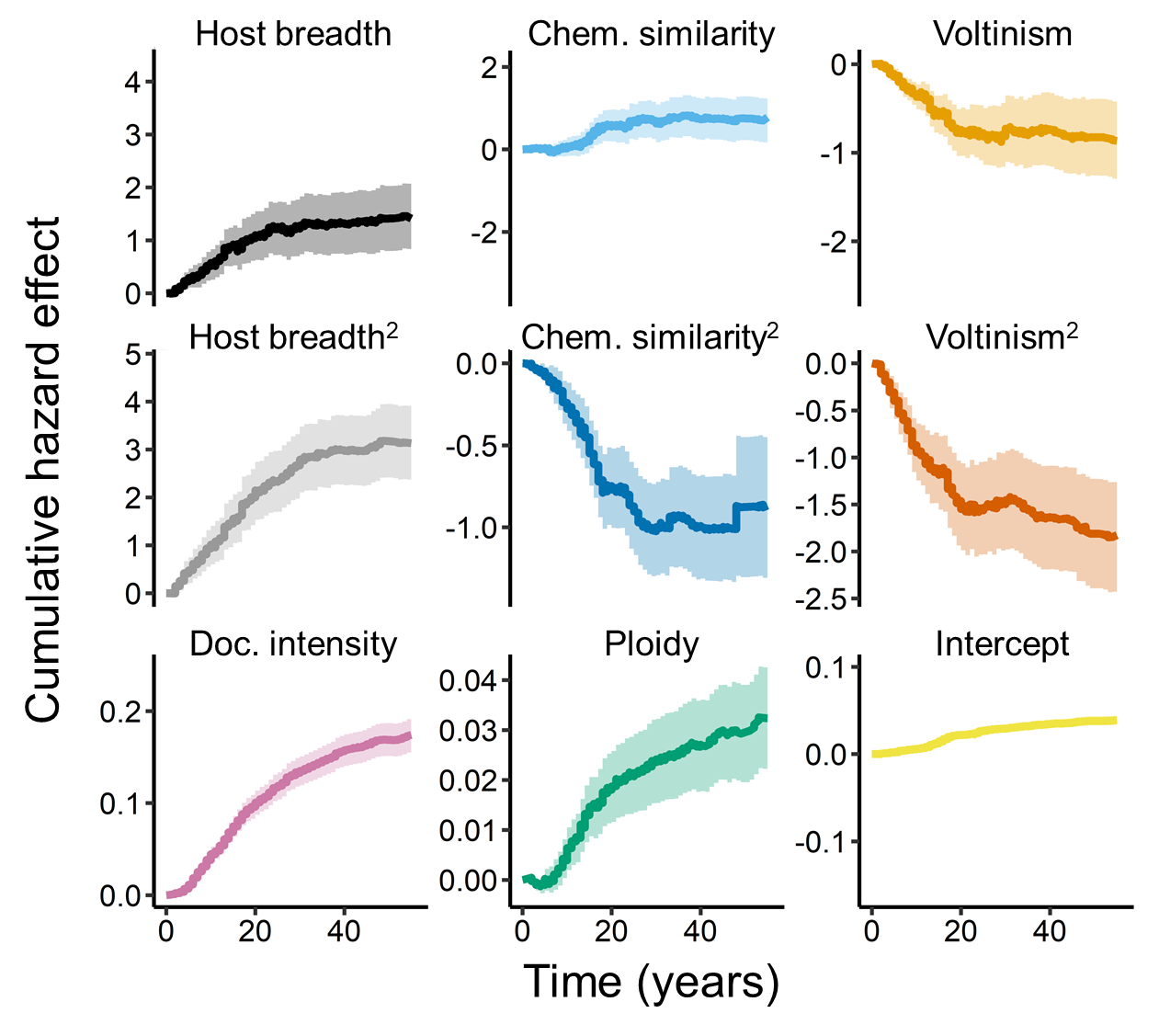
**

**Supplementary Figure 2.** **Effects of predictors on probability of insecticide resistance evolution** **over time.** Line plots depict cumulative hazard effects from all-in additive hazard model (light shading represents 95% confidence intervals). The intercept plot shows how the baseline risk of insecticide resistance increases over time. The rest of the plots show how each model covariate modify that baseline hazard.

**
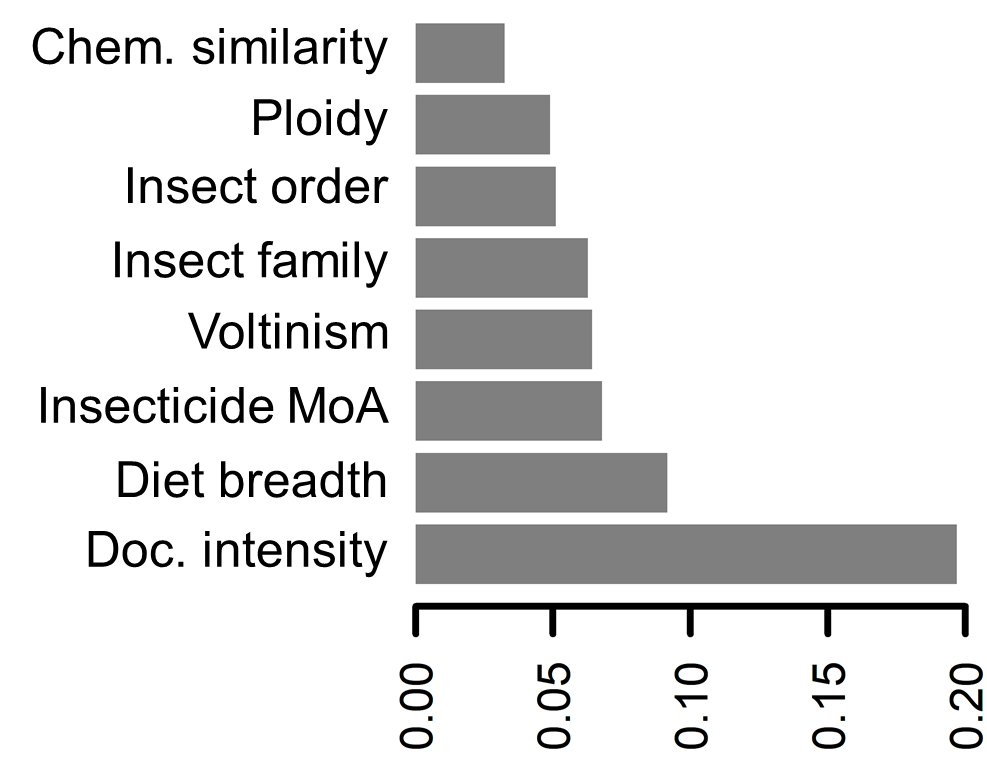
**

**Supplementary Figure 3.** **All-in Random Forest survival model variable importance**.
